# Mast Cells, Neuroinflammation and Pain in Fibromyalgia Syndrome

**DOI:** 10.3389/fncel.2019.00353

**Published:** 2019-08-02

**Authors:** Theoharis C. Theoharides, Irene Tsilioni, Mona Bawazeer

**Affiliations:** ^1^Laboratory of Molecular Immunopharmacology and Drug Discovery, Department of Immunology, Tufts University School of Medicine, Boston, MA, United States; ^2^Sackler School of Graduate Biomedical Sciences, Tufts University, Boston, MA, United States; ^3^Department of Internal Medicine, Tufts Medical Center, Tufts University School of Medicine, Boston, MA, United States; ^4^Department of Psychiatry, Tufts Medical Center, Tufts University School of Medicine, Boston, MA, United States; ^5^Department of Basic Medical Sciences, College of Medicine, King Saud Bin Abdulaziz University for Health Sciences, Riyadh, Saudi Arabia

**Keywords:** mast cells, pain, neuroinflammation, fibromyalgia syndrome, proinflammatory cytokines (TNF-alpha, IL-1 beta, IL-6)

## Abstract

Fibromyalgia Syndrome (FMS) is a disorder of chronic, generalized muscular pain, accompanied by sleep disturbances, fatigue and cognitive dysfunction. There is no definitive pathogenesis except for altered central pain pathways. We previously reported increased serum levels of the neuropeptides substance P (SP) and its structural analogue hemokinin-1 (HK-1) together with the pro-inflammatory cytokines IL-6 and TNF in FMS patients as compared to sedentary controls. We hypothesize that thalamic mast cells contribute to inflammation and pain, by releasing neuro-sensitizing molecules that include histamine, IL-1β, IL-6 and TNF, as well as calcitonin-gene related peptide (CGRP), HK-1 and SP. These molecules could either stimulate thalamic nociceptive neurons directly, or via stimulation of microglia in the diencephalon. As a result, inhibiting mast cell stimulation could be used as a novel approach for reducing pain and the symptoms of FMS.

## Introduction

Fibromyalgia Syndrome is a disorder of chronic generalized muscular pain, stiffness, generalized fatigue, sleep abnormalities, (Clauw et al., 2011; Schmidt-Wilcke and Clauw, 2011; Clauw, 2014) and cognitive problems (Theoharides et al., 2015b; Hauser et al., 2019) assessed by the FSQ (Ferrari and Russell, 2013), which has about 93% sensitivity and 92% specificity (Clauw, 2014). FMS affects about 5% of adults, primarily women 20–60 years of age (Branco et al., 2010) and belongs to a family of overlapping painful conditions (Table 1) known as CSS (Yunus, 2007; Theoharides, 2013). Central sensitization is recognized as the main mechanism involved (Woodman, 2013) and is characterized by allodynia, pain from an otherwise non-painful stimulus, (Russell and Larson, 2009) and hyperalgesia (Staud et al., 2001) due to an exaggerated response to a painful stimulus (Woolf, 2011). The pathogenesis of FMS remains unknown and with no objective diagnostic criteria (McBeth and Mulvey, 2012; Wolfe and Walitt, 2013). FMS patients have reduced tolerance to pain, especially extremes of heat and cold (Desmeules et al., 2003). There is considerable evidence of altered circuity of pain networks and (Jensen et al., 2012; Flodin et al., 2014) abnormal pain processing in FMS (Staud, 2011).

**TABLE 1:** Pain Syndromes Comorbid with FMS.

• Chronic inflammatory response syndrome (CIRS) • Functional dyspepsia • Gulf War Illness (GWI) • Interstitial cystitis/bladder pain syndrome (IC/BPS) • Irritable bowel syndrome (IBS) • Mast cell mediator disorder (MCMD) • Mastocytosis • Migraines • Myalgic Encephalomyelitis/Chronic Fatigue Syndrome (ME/CFS) • Myogenic temporomandibular disorder (TMD) • Myofacial pain syndrome • Post-traumatic stress disorder (PTSD) • Restless leg syndrome • Temporomandibular pain syndrome (TMS) • Tension headache

The PubMed database was searched between 1960 and 2018 using the terms fatigue, fibromyalgia, hypothalamus, inflammation, mast cells, pain and stress. Only articles in English were included.

Here we discuss how brain mast cell release of neuro-sensitizing mediators in the thalamus leads to focal inflammation and contribute to the pathogenesis of FMS.

### Neurohormonal Triggers of Mast Cells Contribute to Focal Inflammation in the Diencephalon

It was recently proposed that FMS may involve localized inflammation in the hypothalamus (Theoharides et al., 2015c). Elevations in pro-inflammatory chemokines/cytokines could negatively impact symptoms (Bazzichi et al., 2007; Carvalho et al., 2008; Nugraha et al., 2013) leading to sensitization of peripheral and central nociceptors (Uceyler et al., 2011; Behm et al., 2012; Hornig et al., 2015). Increased levels of the pro-inflammatory chemokine IL-8 (CXCL8) have been reported in the serum and CSF in patients with FMS (Ross et al., 2010; Kadetoff et al., 2012; Rodriguez-Pinto et al., 2014). Chemokines facilitate nociception by directly acting on chemokine receptors present along the pain pathway (Abbadie, 2005; Charo and Ransohoff, 2006).

The cytokines TNF and IL-17 greatly contribute to the inflammatory response (Romero-Sanchez et al., 2011; Griffin et al., 2012). Plasma levels of IL-17 were increased and correlated with levels of TNF in patients with FMS (Pernambuco et al., 2013). CSF and serum IL-17 also positively correlated with pain (Meng et al., 2013) and anxiety (Liu et al., 2012). Mast cells, themselves, can secrete IL-17; moreover, IL-6 and TGFβ from mast cells contribute to the development of Th-17 cells (Kenna and Brown, 2013).

Fibromyalgia syndrome worsen by stress, (Geenen et al., 2002) which augments pain responses (Bote et al., 2012, 2013). Plasma concentrations of cortisol are increased in the evening, suggesting disruption of the circadian rhythm (Crofford et al., 2004). Serum levels of CRH, which is secreted under stress, were increased in patients with FMS (Tsilioni et al., 2016). CRH was also increased in the CSF of such patients and correlated with severity of pain (McLean et al., 2006). Physiological stress was reported to be the most common trigger in patients with systemic mastocytosis (SM) (Jennings et al., 2014) who also commonly experience FMS (Theoharides et al., 2015d, 2019). We reported increased levels of CRH in the serum of one patient with indolent systemic mastocytosis (Theoharides et al., 2014). CRH can trigger human mast cells to release VEGF without histamine or tryptase (Cao et al., 2005). CRH also has synergistic action with NT stimulating VEGF release. As a result, there is increased vascular permeability in the skin and the blood-brain barrier (BBB) (Esposito et al., 2002; Donelan et al., 2006; Theoharides and Konstantinidou, 2007). Stress also disrupts the gut-blood barrier (Theoharides et al., 1999; Wallon et al., 2008) allowing for gut microbiome-associated molecules, such as propionate (Minerbi et al., 2019) to enter the brain and contribute to focal inflammation. These results have led to the conclusion that mast cells may serve as “immune gate to the brain” (Theoharides, 1990; Ribatti, 2015).

Levels of the neuropeptide SP (Russell, 1998) and NGF (Giovengo et al., 1999) are elevated in the CSF of FMS patients. NGF has been reported to increase nociception and hyperalgesia (Maren, 2017). The SP receptor NK-1 has been involved in the pathophysiology of pain (Greenwood-Van et al., 2014). We reported increased serum levels of SP, its structural analogue Hemokinin-1 (HK-1) and TNF in patients with FMS (Tsilioni et al., 2016). SP (Theoharides et al., 2010a,b) and NGF (Levi-Montalcini, 1987) can stimulate mast cells. Moreover, SP induced mast cell expression of CRHR-1 (Scholzen et al., 2001). Cerebrovascular mast cells were stimulated by CGRP, (Reynier-Rebuffel et al., 1994; Ottosson and Edvinsson, 1997) which is now well established to participate in the pathophysiology of headaches (Edvinsson, 2018). In addition to neuropeptides, sex hormones can also affect mast cell reactivity. For instance, estradiol augments immune (Kovats, 2015) and allergic (Hox et al., 2015) processes. In particular, we had reported expression of estrogen receptors on rodent mast cells (Pang et al., 1995). We also reported that 17β-estradiol further increased stimulation of mast cells by SP (Theoharides et al., 1993). Such findings may help explain why FMS is more common in women.

In addition to allergic reactions, mast cells contribute to innate immunity, (Galli et al., 2011) autoimmunity (Rottem and Mekori, 2005) and inflammation (Theoharides et al., 2010a).

### Thalamic Mast Cells Secrete Neurosensitizing Mediators

**FIGURE 1:**
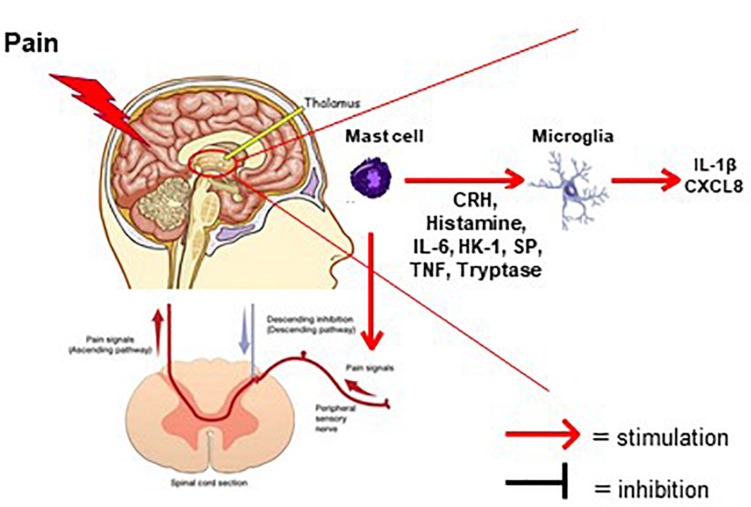
Diagram depicting the involvement of mast cells in the generation of pain in FMS. Mast cells (violet color) in the thalamus secrete pro-inflammatory and neuro-sensitizing mediators (CRH, histamine, IL-6, HK-1, SP, TNF, Tryptase). These mediators can then activate either microglia in thalamic nuclei or ascending nociceptive tracks creating the sensation of pain. Possible natural molecules to inhibit stimulated mast cells and/or microglia are flavonoids such as luteolin or tetramethoxyluteolin (Methlut).

Increasing evidence supports the involvement of mast cells in FMS (Lucas et al., 2006; Pollack, 2014) and comorbid disorders (Theoharides, 2013) as well as other inflammatory (Galli et al., 2008; Theoharides et al., 2010a) and painful conditions, (Heron and Dubayle, 2013; Chatterjea and Martinov, 2014) as well as neuroimmune interactions (Skaper et al., 2017) (Figure 1). Chronic urticaria, which involves stimulation of skin mast cells is more common in FMS (Torresani et al., 2009). Moreover, mast cells are significantly increased in the papillary dermis of FMS patients (Blanco et al., 2010). The chemokines monocyte chemoattractant protein-1 (MCP-1/CCL2) and eotaxin (CCL-11) are elevated in plasma of FMS patients (Zhang et al., 2008). MCP-1 is a strong mast cell chemoattractant (Conti et al., 1998) and also triggers mast cells in rodents (Conti and Theoharides, 1994). MCP-1 induced prolonged muscle hyperalgesia in rats via activation of its high-affinity receptor, CC Chemokine receptor 2 (CCR2), on the peripheral nerve terminals (Alvarez et al., 2014). Myoblasts treated with MCP-1 secreted significant amounts of the key pro-inflammatory cytokine IL-1β (Zhang et al., 2008). C-reactive protein (CRP) is now considered a marker of chronic inflammation. CRP may be useful in the diagnostic of FMS (and depression/anxiety that often accompany FMS), even though there is no direct correlation reported (De Berardis et al., 2006, 2017; Orsolini et al., 2018).

Mast cells derive from the bone marrow and mature in response to SCF, which acts via the cell surface tyrosine kinase KIT receptor (Galli et al., 2011). Mast cell progenitors then migrate in all tissues. As a result, mast cell mediators can affect all organs and lead to multiple symptoms. Mast cells are found adjacent to blood vessels and nerve endings; in the brain, mast cells are located in the thalamus, hypothalamus and median eminence (Edvinsson et al., 1976; Lambracht-Hall et al., 1990; Theoharides et al., 2015d).

Mast cells are known to be stimulated by IgE, via activation of its unique surface receptors (FcεRI) (Rivera et al., 2008). Mast cells can also be stimulated via TLRs, (Abraham and St John, 2010; Zhang et al., 2010). Stimulated mast cells secrete multiple vasoactive, pro-inflammatory and neuro-sensitizing molecules (Galli and Tsai, 2008; Theoharides et al., 2010a). Stimulation of mast cells can be augmented by the cytokine IL-33, (Fux et al., 2014) which synergizes with SP to induce release of impressive amounts of VEGF, (Theoharides et al., 2010b) TNF (Taracanova et al., 2017) or IL-1β (Taracanova et al., 2018). As a result, mast cells can serve as “sensors of cell danger” (Theoharides, 1996; Enoksson et al., 2011; Theoharides et al., 2015a).

Mast cell secretory granules store many preformed pro-inflammatory and neuro-sensitizing mediators including bradykinin, histamine, TNF and tryptase (Nakae et al., 2005; Olszewski et al., 2007). Mast cells also release *de novo* synthesized molecules: (a) lipid mediators (leukotrienes, prostaglandins, and PAF), (b) cytokines (IL-6, IL-13, IL-33, TNF) and (c) chemokines (CXCL8, CCL2, CCL5), (Theoharides et al., 2015d; Mukai et al., 2018). Mast cell could often release mediators selectively without histamine or tryptase (Theoharides et al., 2007). Mast cells also release IL-31, which is important in the sensation of itching and pain, in response to IgE and SP, IL-33 and specifically their combination (Petra et al., 2018). We reported that mast cells can release mtDNA, which is mistaken as a pathogen and stimulates inflammatory responses (Zhang B. et al., 2012).

Finally, mast cells can release extracellular vesicles (exosomes) (Skokos et al., 2002, 2003) that could deliver regulatory molecules, including mtDNA and microRNAs (Kawikova and Askenase, 2014). Such microvesicles have been implicated in brain disorders (Tsilioni et al., 2014; Kawikova and Askenase, 2014) and pain disorders (Rafiee et al., 2018; Silva-Freire et al., 2019). We recently reported that extracellular vesicles are increased in the serum of children with ASD, contained mtDNA and stimulated cultured human microglia to secrete the pro-inflammatory molecules IL-1β and CXCL8 (Tsilioni and Theoharides, 2018).

### Mast Cell Interactions With Microglia

Mast cells communicate with microglia (Skaper et al., 2012, 2014b). Mediators secreted from mast cells, (Zhang et al., 2016) such as histamine (Dong et al., 2014) and tryptase, (Zhang S. et al., 2012) can activate microglia leading to secretion of the pro-inflammatory cytokines IL-1β, IL-6 and TNF. Microglia can also be activated by CRH secreted from mast cells (Wang et al., 2002; Kempuraj et al., 2004). Stimulation of brain mast cells in mice led to activation of microglia, which was decreased by administration of a mast cell inhibitor (Dong et al., 2017).

Microglia are involved in synapse plasticity, (Shemer et al., 2015; Wu et al., 2015; Reu et al., 2017) but are responsible for innate immunity of the brain (Ransohoff and Brown, 2012; Aguzzi et al., 2013). Microglia contribute to brain inflammation (Hagberg et al., 2012; Aguzzi et al., 2013; Nakagawa and Chiba, 2016) and the pathogenesis of different brain disorders, (Takeda et al., 2014; Reus et al., 2015; Faden et al., 2016; Garden and Campbell, 2016; Groh and Martini, 2017; Koutsouras et al., 2017; Pennisi et al., 2017; Jiang et al., 2018; Thonhoff et al., 2018) especially ASD (Vargas et al., 2005; Morgan et al., 2010; Suzuki et al., 2013; Edmonson et al., 2014; Gupta et al., 2014). Microglia in the thalamus have been discussed in the context of pain, especially maintaining the pain sensation even after the original painful stimulus is not present (Banati, 2002; Hansson, 2010; Saghaei et al., 2013; Blaszczyk et al., 2018).

## Conclusion

Mast cells have been implicated in headaches (Theoharides, 1983; Theoharides et al., 2005) and pain (Xanthos et al., 2011; Aich et al., 2015; Gupta and Harvima, 2018). Activation of the mast cell-specific receptor, MRGPRX2, (McNeil et al., 2015) and its mouse analogue, Mrgprb2, mediated inflammatory mechanical and thermal hyperalgesia (Green et al., 2019). Hence, mast cells are key players of neuroendocrine (Theoharides, 2017) and painful disorders (Theoharides et al., 2019).

In this context, inhibitors of mast cells (Harvima et al., 2014) would be useful in the treatment of FMS. Natural molecules could include the flavonoids, luteolin (Kempuraj et al., 2008; Theoharides et al., 2015c; Ashaari et al., 2018) and tetramethoxyluteolin, (Theoharides et al., 2017; Theoharides and Tsilioni, 2018) alone or in combination with other substances selected to reduce stress (Theoharides and Kavalioti, 2018). Other natural molecules could include palmitoylethanolamide, (Schweiger et al., 2019) which apparently inhibits neuro-inflammation (Skaper et al., 2013, 2015) and reduces pain (Skaper et al., 2014a; Impellizzeri et al., 2016).

## Future Directions

Research should focus on identifying in serum of patients with FMS novel molecules that are involved in pain transmission such as bradykinin, CGRP and IL-31. Extracellular vesicles should also be isolated from the serum and CSF of FMS patients, their content identified, and their effect investigated on cultured human mast cells and microglia. Such possible interactions would serve as useful *in vitro* assays for the screening of potential novel treatment agents. Recent reports have also stressed the possible use of the cytokine IL-37, (Mastrangelo et al., 2018) which is known to have anti-inflammatory actions (Cavalli and Dinarello, 2018). It would be important to explore the possible use of IL-37 isoforms in the treatment of FMS.

## Author Contributions

TT, IT, and MB participated in searching the literature. TT and IT wrote or contributed to the writing of the manuscript. IT prepared the figure.

## Conflict of Interest Statement

TT is the inventor of US patents No. 7,906,153 and No. 8,268,365 for the treatment of neuroinflammatory conditions. The remaining authors declare that the research was conducted in the absence of any commercial or financial relationships that could be construed as a potential conflict of interest.

## Abbreviations

ACR: American College of Rheumatology
ASD: autism spectrum disorder
CGRP: calcitonin-gene related peptide
CIRS: chronic inflammatory response syndrome
CRH: corticotropin-releasing hormone
CSF: cerebrospinal fluid
CSS: central sensitivity syndromes
CXCL8: IL-8
FcεRI: high affinity surface receptors
FMS: fibromyalgia syndrome
FSQ: fibromyalgia survey questionnaire
IBS: irritable bowel syndrome
IC/BPS: interstitial cystitis/bladder pain syndrome
IgE: immunoglobulin E
MCP-1/CCL2: monocyte chemoattractant protein-1
ME/CFS: myalgic encephalomyelitis/chronic fatigue syndrome
MRGPRX2: mas-related G-protein coupled receptor member X2
(mt)DNA: mitochondrial
NGF: nerve growth factor
PAF: platelet activating factor
PTSD: post-traumatic stress disorder
SCF: stem cell factor
SP: substance P
TLRs: toll-like receptors
TMD: myogenic temporomandibular disorder.

## References

[B1] Abbadie C. (2005). Chemokines, chemokine receptors and pain. *Trends Immunol.* 26 529–534. 10.1016/j.it.2005.08.001 16099720

[B2] Abraham S. N. St John A. L. (2010). Mast cell-orchestrated immunity to pathogens. *Nat. Rev. Immunol.* 10 440–452. 10.1038/nri2782 20498670 PMC4469150

[B3] Aguzzi A. Barres B. A. Bennett M. L. (2013). Microglia: scapegoat, saboteur, or something else? *Science* 339 156–161. 10.1126/science.1227901 23307732 PMC4431634

[B4] Aich A. Afrin L. B. Gupta K. (2015). Mast cell-mediated mechanisms of nociception. *Int. J. Mol. Sci.* 16 29069–29092. 10.3390/ijms161226151 26690128 PMC4691098

[B5] Alvarez P. Green P. G. Levine J. D. (2014). Role for monocyte chemoattractant protein-1 in the induction of chronic muscle pain in the rat. *Pain* 155 1161–1167. 10.1016/j.pain.2014.03.004 24637038 PMC4303347

[B6] Ashaari Z. Hadjzadeh M. A. Hassanzadeh G. Alizamir T. Yousefi B. Keshavarzi Z. (2018). The flavone luteolin improves central nervous system disorders by different mechanisms: a review. *J. Mol. Neurosci.* 65 491–506. 10.1007/s12031-018-1094-2 30083786

[B7] Banati R. B. (2002). Brain plasticity and microglia: is transsynaptic glial activation in the thalamus after limb denervation linked to cortical plasticity and central sensitisation? *J. Physiol. Paris* 96 289–299. 10.1016/s0928-4257(02)00018-9 12445908

[B8] Bazzichi L. Rossi A. Massimetti G. Giannaccini G. Giuliano T. De Feo F. (2007). Cytokine patterns in fibromyalgia and their correlation with clinical manifestations. *Clin. Exp. Rheumatol.* 25 225–230. 17543146

[B9] Behm F. G. Gavin I. M. Karpenko O. Lindgren V. Gaitonde S. Gashkoff P. A. (2012). Unique immunologic patterns in fibromyalgia. *BMC Clin. Pathol.* 12:25. 10.1186/1472-6890-12-25 23245186 PMC3534336

[B10] Blanco I. Beritze N. Arguelles M. Carcaba V. Fernandez F. Janciauskiene S. (2010). Abnormal overexpression of mastocytes in skin biopsies of fibromyalgia patients. *Clin. Rheumatol.* 29 1403–1412. 10.1007/s10067-010-1474-7 20428906

[B11] Blaszczyk L. Maitre M. Leste-Lasserre T. Clark S. Cota D. Oliet S. H. R. (2018). Sequential alteration of microglia and astrocytes in the rat thalamus following spinal nerve ligation. *J. Neuroinflammation* 15:349. 10.1186/s12974-018-1378-z 30572902 PMC6302506

[B12] Bote M. E. Garcia J. J. Hinchado M. D. Ortega E. (2012). Inflammatory/stress feedback dysregulation in women with fibromyalgia. *Neuroimmunomodulation* 19 343–351. 10.1159/000341664 22986514

[B13] Bote M. E. Garcia J. J. Hinchado M. D. Ortega E. (2013). Fibromyalgia: anti-inflammatory and stress responses after acute moderate exercise. *PLoS One* 8:e74524. 10.1371/journal.pone.0074524 24023948 PMC3762808

[B14] Branco J. C. Bannwarth B. Failde I. Abello C. J. Blotman F. Spaeth M. (2010). Prevalence of fibromyalgia: a survey in five European countries. *Semin. Arthritis Rheum.* 39 448–453. 10.1016/j.semarthrit.2008.12.003 19250656

[B15] Cao J. Papadopoulou N. Kempuraj D. Boucher W. S. Sugimoto K. Cetrulo C. L. (2005). Human mast cells express corticotropin-releasing hormone (CRH) receptors and CRH leads to selective secretion of vascular endothelial growth factor. *J. Immunol.* 174 7665–7675. 10.4049/jimmunol.174.12.7665 15944267

[B16] Carvalho L. S. Correa H. Silva G. C. Campos F. S. Baiao F. R. Ribeiro L. S. (2008). May genetic factors in fibromyalgia help to identify patients with differentially altered frequencies of immune cells? *Clin. Exp. Immunol.* 154 346–352. 10.1111/j.1365-2249.2008.03787.x 19037919 PMC2633236

[B17] Cavalli G. Dinarello C. A. (2018). Suppression of inflammation and acquired immunity by IL-37. *Immunol. Rev.* 281 179–190. 10.1111/imr.12605 29247987

[B18] Charo I. F. Ransohoff R. M. (2006). The many roles of chemokines and chemokine receptors in inflammation. *N. Engl. J. Med.* 354 610–621. 10.1056/nejmra052723 16467548

[B19] Chatterjea D. Martinov T. (2014). Mast cells: versatile gatekeepers of pain. *Mol. Immunol.* 63 38–44. 10.1016/j.molimm.2014.03.001 24666768 PMC4171343

[B20] Clauw D. J. (2014). Fibromyalgia: a clinical review. *JAMA* 311 1547–1555. 10.1001/jama.2014.3266 24737367

[B21] Clauw D. J. Arnold L. M. McCarberg B. H. (2011). The science of fibromyalgia. *Mayo Clin. Proc.* 86 907–911. 10.4065/mcp.2011.0206 21878603 PMC3258006

[B22] Conti P. Reale M. Barbacane R. C. Letourneau R. Theoharides T. C. (1998). Intramuscular injection of hrRANTES causes mast cell recruitment and increased transcription of histidine decarboxylase: lack of effects in genetically mast cell-deficient W/Wv mice. *FASEB J.* 12 1693–1700. 10.1096/fasebj.12.15.1693 9837859

[B23] Conti P. Theoharides T. C. (1994). Monocyte chemotactic Protein-1 (MCP-1) is active on mast cells and causes clump formation. *Int. J. Immunopathol. Pharmacol.* 7 149–151. 8550082

[B24] Crofford L. J. Young E. A. Engleberg N. C. Korszun A. Brucksch C. B. McClure L. A. (2004). Basal circadian and pulsatile ACTH and cortisol secretion in patients with fibromyalgia and/or chronic fatigue syndrome. *Brain Behav. Immun.* 18 314–325. 10.1016/s0889-1591(04)00021-2 15157948

[B25] De Berardis D. Campanella D. Gambi F. La R. R. Carano A. Conti C. M. (2006). The role of C-reactive protein in mood disorders. *Int. J. Immunopathol. Pharmacol.* 19 721–725. 17166394 10.1177/039463200601900402

[B26] De Berardis D. Serroni N. Campanella D. Marini S. Rapini G. Valchera A. (2017). Alexithymia, suicide ideation, C-Reactive Protein, and serum lipid levels among outpatients with generalized anxiety disorder. *Arch. Suicide Res.* 21 100–112. 10.1080/13811118.2015.1004485 25856390

[B27] Desmeules J. A. Cedraschi C. Rapiti E. Baumgartner E. Finckh A. Cohen P. (2003). Neurophysiologic evidence for a central sensitization in patients with fibromyalgia. *Arthritis Rheum.* 48 1420–1429. 10.1002/art.10893 12746916

[B28] Donelan J. Boucher W. Papadopoulou N. Lytinas M. Papaliodis D. Theoharides T. C. (2006). Corticotropin-releasing hormone induces skin vascular permeability through a neurotensin-dependent process. *Proc. Natl. Acad. Sci. U.S.A.* 103 7759–7764. 10.1073/pnas.0602210103 16682628 PMC1472518

[B29] Dong H. Zhang W. Zeng X. Hu G. Zhang H. He S. (2014). Histamine induces upregulated expression of histamine receptors and increases release of inflammatory mediators from microglia1. *Mol. Neurobiol.* 49 1487–1500. 10.1007/s12035-014-8697-6 24752587

[B30] Dong H. Zhang X. Wang Y. Zhou X. Qian Y. Zhang S. (2017). Suppression of brain mast cells degranulation inhibits microglial activation and central nervous system inflammation. *Mol. Neurobiol.* 54 997–1007. 10.1007/s12035-016-9720-x 26797518

[B31] Edmonson C. Ziats M. N. Rennert O. M. (2014). Altered glial marker expression in autistic post-mortem prefrontal cortex and cerebellum. *Mol. Autism* 5:3. 10.1186/2040-2392-5-3 24410870 PMC3914711

[B32] Edvinsson L. (2018). The CGRP pathway in migraine as a viable target for therapies. *Headache* 58(Suppl. 1), 33–47. 10.1111/head.13305 29697153

[B33] Edvinsson L. Owman C. Sjöberg N. O. (1976). Autonomic nerves, mast cells and amine receptors in human brain vessels. A histochemical and pharmacological study. *Brain Res.* 115 377–393. 10.1016/0006-8993(76)90356-5 184880

[B34] Enoksson M. Lyberg K. Moller-Westerberg C. Fallon P. G. Nilsson G. Lunderius-Andersson C. (2011). Mast cells as sensors of cell injury through IL-33 recognition. *J. Immunol.* 186 2523–2528. 10.4049/jimmunol.1003383 21239713

[B35] Esposito P. Chandler N. Kandere-Grzybowska K. Basu S. Jacobson S. Connolly R. (2002). Corticotropin-releasing hormone (CRH) and brain mast cells regulate blood-brain-barrier permeability induced by acute stress. *J. Pharmacol. Exp. Ther.* 303 1061–1066. 10.1124/jpet.102.038497 12438528

[B36] Faden A. I. Wu J. Stoica B. A. Loane D. J. (2016). Progressive inflammation-mediated neurodegeneration after traumatic brain or spinal cord injury. *Br. J. Pharmacol.* 173 681–691. 10.1111/bph.13179 25939377 PMC4742301

[B37] Ferrari R. Russell A. S. (2013). A questionnaire using the modified 2010 American College of rheumatology criteria for fibromyalgia: specificity and sensitivity in clinical practice. *J. Rheumatol.* 40 1590–1595. 10.3899/jrheum.130367 23818707

[B38] Flodin P. D. Martinsen S. Lofgren M. Bileviciute-Ljungar I. Kosek E. Fransson P. (2014). Fibromyalgia is associated with decreased connectivity between pain- and sensorimotor brain areas. *Brain Connect.* 4 587–594. 10.1089/brain.2014.0274 24998297 PMC4202907

[B39] Fux M. Pecaric-Petkovic T. Odermatt A. Hausmann O. V. Lorentz A. Bischoff S. C. (2014). IL-33 is a mediator rather than a trigger of the acute allergic response in humans. *Allergy* 69 216–222. 10.1111/all.12309 24205920

[B40] Galli S. J. Borregaard N. Wynn T. A. (2011). Phenotypic and functional plasticity of cells of innate immunity: macrophages, mast cells and neutrophils. *Nat. Immunol.* 12 1035–1044. 10.1038/ni.2109 22012443 PMC3412172

[B41] Galli S. J. Tsai M. (2008). Mast cells: versatile regulators of inflammation, tissue remodeling, host defense and homeostasis. *J. Dermatol. Sci.* 49 7–19. 10.1016/j.jdermsci.2007.09.009 18024086 PMC2788430

[B42] Galli S. J. Tsai M. Piliponsky A. M. (2008). The development of allergic inflammation. *Nature* 454 445–454. 10.1038/nature07204 18650915 PMC3573758

[B43] Garden G. A. Campbell B. M. (2016). Glial biomarkers in human central nervous system disease. *Glia* 64 1755–1771. 10.1002/glia.22998 27228454 PMC5575821

[B44] Geenen R. Jacobs J. W. Bijlsma J. W. (2002). Evaluation and management of endocrine dysfunction in fibromyalgia. *Rheum. Dis. Clin. North Am.* 28 389–404. 10.1016/s0889-857x(01)00009-6 12122926

[B45] Giovengo S. L. Russell I. J. Larson A. A. (1999). Increased concentrations of nerve growth factor in cerebrospinal fluid of patients with fibromyalgia. *J. Rheumatol.* 26 1564–1569. 10405946

[B46] Green D. P. Limjunyawong N. Gour N. Pundir P. Dong X. (2019). A Mast-Cell-Specific receptor mediates neurogenic inflammation and pain. *Neuron* 101 412–420. 10.1016/j.neuron.2019.01.012 30686732 PMC6462816

[B47] Greenwood-Van M. B. Mohammadi E. Tyler K. Pietra C. Bee L. A. Dickenson A. (2014). Synergistic effect of 5-hydroxytryptamine 3 and neurokinin 1 receptor antagonism in rodent models of somatic and visceral pain. *J. Pharmacol. Exp. Ther.* 351 146–152. 10.1124/jpet.114.216028 25077526

[B48] Griffin G. K. Newton G. Tarrio M. L. Bu D. X. Maganto-Garcia E. Azcutia V. (2012). IL-17 and TNF-alpha sustain neutrophil recruitment during inflammation through synergistic effects on endothelial activation. *J. Immunol.* 188 6287–6299. 10.4049/jimmunol.1200385 22566565 PMC3370121

[B49] Groh J. Martini R. (2017). Neuroinflammation as modifier of genetically caused neurological disorders of the central nervous system: understanding pathogenesis and chances for treatment. *Glia* 65 1407–1422. 10.1002/glia.23162 28568966

[B50] Gupta K. Harvima I. T. (2018). Mast cell-neural interactions contribute to pain and itch. *Immunol. Rev.* 282 168–187. 10.1111/imr.12622 29431216 PMC5812374

[B51] Gupta S. Ellis S. E. Ashar F. N. Moes A. Bader J. S. Zhan J. (2014). Transcriptome analysis reveals dysregulation of innate immune response genes and neuronal activity-dependent genes in autism. *Nat. Commun.* 5:5748. 10.1038/ncomms6748 25494366 PMC4270294

[B52] Hagberg H. Gressens P. Mallard C. (2012). Inflammation during fetal and neonatal life: implications for neurologic and neuropsychiatric disease in children and adults. *Ann. Neurol.* 71 444–457. 10.1002/ana.22620 22334391

[B53] Hansson E. (2010). Long-term pain, neuroinflammation and glial activation. *Scand. J. Pain* 1 67–72. 10.1016/j.sjpain.2010.01.002 29913949

[B54] Harvima I. T. Levi-Schaffer F. Draber P. Friedman S. Polakovicova I. Gibbs B. F. (2014). Molecular targets on mast cells and basophils for novel therapies. *J. Allergy Clin. Immunol.* 134 530–544. 10.1016/j.jaci.2014.03.007 24767877

[B55] Hauser W. Sarzi-Puttini P. Fitzcharles M. A. (2019). Fibromyalgia syndrome: under-, over- and misdiagnosis. *Clin. Exp. Rheumatol.* 37(Suppl. 116), 90–97. 30747096

[B56] Heron A. Dubayle D. (2013). A focus on mast cells and pain. *J. Neuroimmunol.* 264 1–7. 10.1016/j.jneuroim.2013.09.018 24125568

[B57] Hornig M. Montoya J. G. Klimas N. G. Levine S. Felsenstein D. Bateman L. (2015). Distinct plasma immune signatures in ME/CFS are present early in the course of illness. *Sci. Adv.* 1:e1400121. 10.1126/sciadv.1400121 26079000 PMC4465185

[B58] Hox V. Desai A. Bandara G. Gilfillan A. M. Metcalfe D. D. Olivera A. (2015). Estrogen increases the severity of anaphylaxis in female mice through enhanced endothelial nitric oxide synthase expression and nitric oxide production. *J. Allergy Clin. Immunol.* 135 729–736. 10.1016/j.jaci.2014.11.003 25553642 PMC5586107

[B59] Impellizzeri D. Di P. R. Cordaro M. Gugliandolo E. Casili G. Morittu V. M. (2016). Adelmidrol, a palmitoylethanolamide analogue, as a new pharmacological treatment for the management of acute and chronic inflammation. *Biochem. Pharmacol.* 119 27–41. 10.1016/j.bcp.2016.09.001 27599446

[B60] Jennings S. Russell N. Jennings B. Slee V. Sterling L. Castells M. (2014). The mastocytosis society survey on mast cell disorders: patient experiences and perceptions. *J. Allergy Clin. Immunol. Pract.* 2 70–76. 10.1016/j.jaip.2013.09.004 24565772

[B61] Jensen K. B. Loitoile R. Kosek E. Petzke F. Carville S. Fransson P. (2012). Patients with fibromyalgia display less functional connectivity in the brain’s pain inhibitory network. *Mol. Pain* 8:32. 10.1186/1744-8069-8-32 22537768 PMC3404927

[B62] Jiang N. M. Cowan M. Moonah S. N. Petri W. A. Jr. (2018). The impact of systemic inflammation on neurodevelopment. *Trends Mol. Med.* 24 794–804. 10.1016/j.molmed.2018.06.008 30006148 PMC6110951

[B63] Kadetoff D. Lampa J. Westman M. Andersson M. Kosek E. (2012). Evidence of central inflammation in fibromyalgia-increased cerebrospinal fluid interleukin-8 levels. *J. Neuroimmunol.* 242 33–38. 10.1016/j.jneuroim.2011.10.013 22126705

[B64] Kawikova I. Askenase P. W. (2014). Diagnostic and therapeutic potentials of exosomes in CNS diseases. *Brain Res.* 1617 63–71. 10.1016/j.brainres.2014.09.070 25304360 PMC4862949

[B65] Kempuraj D. Papadopoulou N. G. Lytinas M. Huang M. Kandere-Grzybowska K. Madhappan B. (2004). Corticotropin-releasing hormone and its structurally related urocortin are synthesized and secreted by human mast cells. *Endocrinology* 145 43–48. 10.1210/en.2003-0805 14576187

[B66] Kempuraj D. Tagen M. Iliopoulou B. P. Clemons A. Vasiadi M. Boucher W. (2008). Luteolin inhibits myelin basic protein-induced human mast cell activation and mast cell dependent stimulation of Jurkat T cells. *Br. J. Pharmacol.* 155 1076–1084. 10.1038/bjp.2008.356 18806808 PMC2597265

[B67] Kenna T. J. Brown M. A. (2013). The role of IL-17-secreting mast cells in inflammatory joint disease. *Nat. Rev. Rheumatol.* 9 375–379. 10.1038/nrrheum.2012.205 23229447

[B68] Koutsouras G. W. Ramos R. L. Martinez L. R. (2017). Role of microglia in fungal infections of the central nervous system. *Virulence* 8 705–718. 10.1080/21505594.2016.1261789 27858519 PMC5626199

[B69] Kovats S. (2015). Estrogen receptors regulate innate immune cells and signaling pathways. *Cell Immunol.* 294 63–69. 10.1016/j.cellimm.2015.01.018 25682174 PMC4380804

[B70] Lambracht-Hall M. Dimitriadou V. Theoharides T. C. (1990). Migration of mast cells in the developing rat brain. *Dev. Brain Res.* 56 151–159. 10.1016/0165-3806(90)90077-c 2261679

[B71] Levi-Montalcini R. (1987). The nerve growth factor 35 years later. *Science* 237 1154–1162. 10.1126/science.3306916 3306916

[B72] Liu Y. Ho R. C. Mak A. (2012). The role of interleukin (IL)-17 in anxiety and depression of patients with rheumatoid arthritis. *Int. J. Rheum. Dis.* 15 183–187. 10.1111/j.1756-185X.2011.01673.x 22462422

[B73] Lucas H. J. Brauch C. M. Settas L. Theoharides T. C. (2006). Fibromyalgia–new concepts of pathogenesis and treatment. *Int. J. Immunopathol. Pharmacol.* 19 5–10. 16569342

[B74] Maren S. (2017). Synapse-Specific encoding of fear memory in the amygdala. *Neuron* 95 988–990. 10.1016/j.neuron.2017.08.020 28858626

[B75] Mastrangelo F. Frydas I. Ronconi G. Kritas S. K. Tettamanti L. Caraffa A. (2018). Low-grade chronic inflammation mediated by mast cells in fibromyalgia: role of IL-37. *J. Biol. Regul. Homeost Agents* 32 195–198. 29684996

[B76] McBeth J. Mulvey M. R. (2012). Fibromyalgia: mechanisms and potential impact of the ACR 2010 classification criteria. *Nat. Rev. Rheumatol.* 8 108–116. 10.1038/nrrheum.2011.216 22270077

[B77] McLean S. A. Williams D. A. Stein P. K. Harris R. E. Lyden A. K. Whalen G. (2006). Cerebrospinal fluid corticotropin-releasing factor concentration is associated with pain but not fatigue symptoms in patients with fibromyalgia. *Neuropsychopharmacology* 31 2776–2782. 10.1038/sj.npp.1301200 16936702 PMC4831068

[B78] McNeil B. D. Pundir P. Meeker S. Han L. Undem B. J. Kulka M. (2015). Identification of a mast-cell-specific receptor crucial for pseudo-allergic drug reactions. *Nature* 519 237–241. 10.1038/nature14022 25517090 PMC4359082

[B79] Meng X. Zhang Y. Lao L. Saito R. Li A. Backman C. M. (2013). Spinal interleukin-17 promotes thermal hyperalgesia and NMDA NR1 phosphorylation in an inflammatory pain rat model. *Pain* 154 294–305. 10.1016/j.pain.2012.10.022 23246025 PMC3563420

[B80] Minerbi A. Gonzalez E. Brereton N. J. B. Anjarkouchian A. Dewar K. Fitzcharles M. A. (2019). Altered microbiome composition in individuals with fibromyalgia. *Pain* 10.1097/j.pain.0000000000001640 [Epub ahead of print]. 31219947

[B81] Morgan J. T. Chana G. Pardo C. A. Achim C. Semendeferi K. Buckwalter J. (2010). Microglial activation and increased microglial density observed in the dorsolateral prefrontal cortex in autism. *Biol. Psychiatry* 68 368–376. 10.1016/j.biopsych.2010.05.024 20674603

[B82] Mukai K. Tsai M. Saito H. Galli S. J. (2018). Mast cells as sources of cytokines, chemokines, and growth factors. *Immunol. Rev.* 282 121–150. 10.1111/imr.12634 29431212 PMC5813811

[B83] Nakae S. Suto H. Kakurai M. Sedgwick J. D. Tsai M. Galli S. J. (2005). Mast cells enhance T cell activation: importance of mast cell-derived TNF. *Proc. Natl. Acad. Sci. U.S.A.* 102 6467–6472. 10.1073/pnas.0501912102 15840716 PMC1088381

[B84] Nakagawa Y. Chiba K. (2016). Involvement of neuroinflammation during brain development in social cognitive deficits in autism spectrum disorder and schizophrenia. *J. Pharmacol. Exp. Ther.* 358 504–515. 10.1124/jpet.116.234476 27384073

[B85] Nugraha B. Korallus C. Kielstein H. Gutenbrunner C. (2013). CD3+CD56+natural killer T cells in fibromyalgia syndrome patients: association with the intensity of depression. *Clin. Exp. Rheumatol.* 31 S9–S15. 23557873

[B86] Olszewski M. B. Groot A. J. Dastych J. Knol E. F. (2007). TNF trafficking to human mast cell granules: mature chain-dependent endocytosis. *J. Immunol.* 178 5701–5709. 10.4049/jimmunol.178.9.5701 17442953

[B87] Orsolini L. Sarchione F. Vellante F. Fornaro M. Matarazzo I. Martinotti G. (2018). Protein-C reactive as biomarker predictor of schizophrenia phases of illness? A systematic review. *Curr. Neuropharmacol.* 16 583–606. 10.2174/1570159X16666180119144538 29357805 PMC5997872

[B88] Ottosson A. Edvinsson L. (1997). Release of histamine from dural mast cells by substance P and calcitonin gene-related peptide. *Cephalalgia* 17 166–174. 10.1046/j.1468-2982.1997.1703166.x 9170339

[B89] Pang X. Cotreau-Bibbo M. M. Sant G. R. Theoharides T. C. (1995). Bladder mast cell expression of high affinity estrogen receptors in patients with interstitial cystitis. *Br. J. Urol.* 75 154–161. 10.1111/j.1464-410x.1995.tb07303.x 7850318

[B90] Pennisi M. Crupi R. Di P. R. Ontario M. L. Bella R. Calabrese E. J. (2017). Inflammasomes, hormesis, and antioxidants in neuroinflammation: role of NRLP3 in Alzheimer disease. *J. Neurosci. Res.* 95 1360–1372. 10.1002/jnr.23986 27862176

[B91] Pernambuco A. P. Schetino L. P. Alvim C. C. Murad C. M. Viana R. S. Carvalho L. S. (2013). Increased levels of IL-17A in patients with fibromyalgia. *Clin. Exp. Rheumatol.* 31 S60–S63. 24021410

[B92] Petra A. I. Tsilioni I. Taracanova A. Katsarou-Katsari A. Theoharides T. C. (2018). Interleukin 33 and interleukin 4 regulate interleukin 31 gene expression and secretion from human laboratory of allergic diseases 2 mast cells stimulated by substance P and/or immunoglobulin E. *Allergy Asthma Proc.* 39 153–160. 10.2500/aap.2018.38.4105 29490771 PMC5827155

[B93] Pollack S. (2014). Mast cells in fibromyalgia. *Clin. Exp. Rheumatol.* 33(1 Suppl. 88):S140. 25236596

[B94] Rafiee Z. A. Falahatian M. Alsahebfosoul F. (2018). Serum levels of histamine and diamine oxidase in multiple sclerosis. *Am. J. Clin. Exp. Immunol.* 7 100–105. 30697467 PMC6334194

[B95] Ransohoff R. M. Brown M. A. (2012). Innate immunity in the central nervous system. *J. Clin. Invest.* 122 1164–1171. 10.1172/JCI58644 22466658 PMC3314450

[B96] Reu P. Khosravi A. Bernard S. Mold J. E. Salehpour M. Alkass K. (2017). The lifespan and turnover of microglia in the human brain. *Cell Rep.* 20 779–784. 10.1016/j.celrep.2017.07.004 28746864 PMC5540680

[B97] Reus G. Z. Fries G. R. Stertz L. Badawy M. Passos I. C. Barichello T. (2015). The role of inflammation and microglial activation in the pathophysiology of psychiatric disorders. *Neuroscience* 300 141–154. 10.1016/j.neuroscience.2015.05.018 25981208

[B98] Reynier-Rebuffel A.-M. Mathiau P. Callebert J. Dimitriadou V. Farjaudon N. Kacem K. (1994). Substance P, calcitonin gene-related peptide, and capsaicin release serotonin from cerebrovascular mast cells. *Am. J. Physiol. Regul. Integr. Comp. Physiol.* 267 R1421–R1429. 7526717 10.1152/ajpregu.1994.267.5.R1421

[B99] Ribatti D. (2015). The crucial role of mast cells in blood-brain barrier alterations. *Exp. Cell Res.* 338 119–125. 10.1016/j.yexcr.2015.05.013 26004870

[B100] Rivera J. Fierro N. A. Olivera A. Suzuki R. (2008). New insights on mast cell activation via the high affinity receptor for IgE. *Adv. Immunol.* 98 85–120. 10.1016/S0065-2776(08)00403-3 18772004 PMC2761150

[B101] Rodriguez-Pinto I. Agmon-Levin N. Howard A. Shoenfeld Y. (2014). Fibromyalgia and cytokines. *Immunol. Lett.* 161 200–203. 10.1016/j.imlet.2014.01.009 24462815

[B102] Romero-Sanchez C. Jaimes D. A. Londono J. De Avila J. Castellanos J. E. Bello J. M. (2011). Association between Th-17 cytokine profile and clinical features in patients with spondyloarthritis. *Clin. Exp. Rheumatol.* 29 828–834. 22041179

[B103] Ross R. L. Jones K. D. Bennett R. M. Ward R. L. Druker B. J. Wood L. J. (2010). Preliminary evidence of increased pain and elevated cytokines in fibromyalgia patients with defective growth hormone response to exercise. *Open Immunol. J.* 3 9–18. 10.2174/1874226201003010009 20467575 PMC2868257

[B104] Rottem M. Mekori Y. A. (2005). Mast cells and autoimmunity. *Autoimmun. Rev.* 4 21–27. 10.1016/j.autrev.2004.05.001 15652775

[B105] Russell I. J. (1998). Advances in fibromyalgia: possible role for central neurochemicals. *Am. J. Med. Sci.* 315 377–384. 10.1016/s0002-9629(15)40355-6 9638894

[B106] Russell I. J. Larson A. A. (2009). Neurophysiopathogenesis of fibromyalgia syndrome: a unified hypothesis. *Rheum. Dis. Clin. North Am.* 35 421–435. 10.1016/j.rdc.2009.06.005 19647152

[B107] Saghaei E. Abbaszadeh F. Naseri K. Ghorbanpoor S. Afhami M. Haeri A. (2013). Estradiol attenuates spinal cord injury-induced pain by suppressing microglial activation in thalamic VPL nuclei of rats. *Neurosci. Res.* 75 316–323. 10.1016/j.neures.2013.01.010 23419864

[B108] Schmidt-Wilcke T. Clauw D. J. (2011). Fibromyalgia: from pathophysiology to therapy. *Nat. Rev. Rheumatol.* 7 518–527. 10.1038/nrrheum.2011.98 21769128

[B109] Scholzen T. E. Steinhoff M. Bonaccorsi P. Klein R. Amadesi S. Geppetti P. (2001). Neutral endopeptidase terminates substance P-induced inflammation in allergic contact dermatitis. *J. Immunol.* 166 1285–1291. 10.4049/jimmunol.166.2.1285 11145711

[B110] Schweiger V. Martini A. Bellamoli P. Donadello K. Schievano C. Del B. G. (2019). Ultramicronized palmitoylethanolamide (um-PEA) as add-on treatment in fibromyalgia syndrome (FMS): retrospective observational study on 407 patients. *CNS Neurol. Disord. Drug Targets* 10.2174/1871527318666190227205359 [Epub ahead of print]. 30827269

[B111] Shemer A. Erny D. Jung S. Prinz M. (2015). Microglia plasticity during health and disease: an immunological perspective. *Trends Immunol.* 36 614–624. 10.1016/j.it.2015.08.003 26431939

[B112] Silva-Freire N. Mayado A. Teodosio C. Jara-Acevedo M. Varez-Twose I. Matito A. (2019). Bone marrow mast cell antibody-targetable cell surface protein expression profiles in systemic mastocytosis. *Int. J. Mol. Sci.* 20:E552. 10.3390/ijms20030552 30696068 PMC6387409

[B113] Skaper S. D. Facci L. Barbierato M. Zusso M. Bruschetta G. Impellizzeri D. (2015). N-Palmitoylethanolamine and neuroinflammation: a novel therapeutic strategy of resolution. *Mol. Neurobiol.* 52 1034–1042. 10.1007/s12035-015-9253-8 26055231

[B114] Skaper S. D. Facci L. Fusco M. la Valle M. F. Zusso M. Costa B. (2014a). Palmitoylethanolamide, a naturally occurring disease-modifying agent in neuropathic pain. *Inflammopharmacology* 22 79–94. 10.1007/s10787-013-0191-7 24178954

[B115] Skaper S. D. Facci L. Giusti P. (2014b). Neuroinflammation, microglia and mast cells in the pathophysiology of neurocognitive disorders: a review. *CNS Neurol. Disord. Drug Targets* 13 1654–1666. 10.2174/1871527313666141130224206 25470401

[B116] Skaper S. D. Facci L. Giusti P. (2013). Glia and mast cells as targets for palmitoylethanolamide, an anti-inflammatory and neuroprotective lipid mediator. *Mol. Neurobiol.* 48 340–352. 10.1007/s12035-013-8487-6 23813098

[B117] Skaper S. D. Facci L. Zusso M. Giusti P. (2017). Neuroinflammation, mast cells, and glia: dangerous liaisons. *Neuroscientist* 23 478–498. 10.1177/1073858416687249 29283023

[B118] Skaper S. D. Giusti P. Facci L. (2012). Microglia and mast cells: two tracks on the road to neuroinflammation. *FASEB J.* 26 3103–3117. 10.1096/fj.11-197194 22516295

[B119] Skokos D. Botros H. G. Demeure C. Morin J. Peronet R. Birkenmeier G. (2003). Mast cell-derived exosomes induce phenotypic and functional maturation of dendritic cells and elicit specific immune responses in vivo. *J. Immunol.* 170 3037–3045. 10.4049/jimmunol.170.6.3037 12626558

[B120] Skokos D. Goubran-Botros H. Roa M. Mecheri S. (2002). Immunoregulatory properties of mast cell-derived exosomes. *Mol. Immunol.* 38 1359–1362. 10.1016/s0161-5890(02)00088-3 12217408

[B121] Staud R. (2011). Brain imaging in fibromyalgia syndrome. *Clin. Exp. Rheumatol.* 29 S109–S117. 22243558

[B122] Staud R. Vierck C. J. Cannon R. L. Mauderli A. P. Price D. D. (2001). Abnormal sensitization and temporal summation of second pain (wind-up) in patients with fibromyalgia syndrome. *Pain* 91 165–175. 10.1016/s0304-3959(00)00432-2 11240089

[B123] Suzuki K. Sugihara G. Ouchi Y. Nakamura K. Futatsubashi M. Takebayashi K. (2013). Microglial activation in young adults with autism spectrum disorder. *JAMA Psychiatry* 70 49–58. 23404112 10.1001/jamapsychiatry.2013.272

[B124] Takeda S. Sato N. Morishita R. (2014). Systemic inflammation, blood-brain barrier vulnerability and cognitive/non-cognitive symptoms in Alzheimer disease: relevance to pathogenesis and therapy. *Front. Aging Neurosci.* 6:171. 10.3389/fnagi.2014.00171 25120476 PMC4114193

[B125] Taracanova A. Alevizos M. Karagkouni A. Weng Z. Norwitz E. Conti P. (2017). SP and IL-33 together markedly enhance TNF synthesis and secretion from human mast cells mediated by the interaction of their receptors. *Proc. Natl. Acad. Sci. U.S.A.* 114 E4002–E4009. 10.1073/pnas.1524845114 28461492 PMC5441798

[B126] Taracanova A. Tsilioni I. Conti P. Norwitz E. R. Leeman S. E. Theoharides T. C. (2018). Substance P and IL-33 administered together stimulate a marked secretion of IL-1beta from human mast cells, inhibited by methoxyluteolin. *Proc. Natl. Acad. Sci. U.S.A* 115 E9381–E9390. 10.1073/pnas.1810133115 30232261 PMC6176605

[B127] Theoharides T. C. (1983). Mast cells and migraines. *Perspect. Biol. Med.* 26 672–675. 10.1353/pbm.1983.0028 6353354

[B128] Theoharides T. C. (1990). Mast cells: the immune gate to the brain. *Life Sci.* 46 607–617. 10.1016/0024-3205(90)90129-f 2407920

[B129] Theoharides T. C. (1996). Mast cell: a neuroimmunoendocrine master player. *Int. J. Tissue React.* 18 1–21. 8880375

[B130] Theoharides T. C. (2013). Atopic conditions in search of pathogenesis and therapy. *Clin. Ther.* 35 544–547. 10.1016/j.clinthera.2013.04.002 23642292

[B131] Theoharides T. C. (2017). Neuroendocrinology of mast cells: challenges and controversies. *Exp. Dermatol.* 26 751–759. 10.1111/exd.13288 28094875

[B132] Theoharides T. C. Alysandratos K. D. Angelidou A. Delivanis D. A. Sismanopoulos N. Zhang B. (2010a). Mast cells and inflammation. *Biochim. Biophys. Acta* 1822 21–33. 21185371 10.1016/j.bbadis.2010.12.014 PMC3318920

[B133] Theoharides T. C. Zhang B. Kempuraj D. Tagen M. Vasiadi M. Angelidou A. (2010b). IL-33 augments substance P-induced VEGF secretion from human mast cells and is increased in psoriatic skin. *Proc. Natl. Acad. Sci. U.S.A.* 107 4448–4453. 10.1073/pnas.1000803107 20160089 PMC2840132

[B134] Theoharides T. C. Dimitriadou V. Letourneau R. J. Rozniecki J. J. Vliagoftis H. Boucher W. S. (1993). Synergistic action of estradiol and myelin basic protein on mast cell secretion and brain demyelination: changes resembling early stages of demyelination. *Neuroscience* 57 861–871. 10.1016/0306-4522(93)90030-j 7508580

[B135] Theoharides T. C. Donelan J. Kandere-Grzybowska K. Konstantinidou A. (2005). The role of mast cells in migraine pathophysiology. *Brain Res. Brain Res. Rev.* 49 65–76. 10.1016/j.brainresrev.2004.11.006 15960987

[B136] Theoharides T. C. Kavalioti M. (2018). Stress, inflammation and natural treatments. *J. Biol. Regul. Homeost Agents* 32 1345–1347. 30574737

[B137] Theoharides T. C. Kempuraj D. Tagen M. Conti P. Kalogeromitros D. (2007). Differential release of mast cell mediators and the pathogenesis of inflammation. *Immunol. Rev.* 217 65–78. 10.1111/j.1600-065x.2007.00519.x 17498052

[B138] Theoharides T. C. Konstantinidou A. (2007). Corticotropin-releasing hormone and the blood-brain-barrier. *Front. Biosci.* 12:1615–1628. 10.2741/2174 17127408

[B139] Theoharides T. C. Letourneau R. Patra P. Hesse L. Pang X. Boucher W. (1999). Stress-induced rat intestinal mast cell intragranular activation and inhibitory effect of sulfated proteoglycans. *Dig. Dis. Sci.* 44 87S–93S. 10490045

[B140] Theoharides T. C. Petra A. I. Stewart J. M. Tsilioni I. Panagiotidou S. Akin C. (2014). High serum corticotropin-releasing hormone (CRH) and bone marrow mast cell CRH receptor expression in a mastocytosis patient. *J. Allergy Clin. Immunol.* 134 1197–1199. 10.1016/j.jaci.2014.05.023 24985398

[B141] Theoharides T. C. Petra A. I. Taracanova A. Panagiotidou S. Conti P. (2015a). Targeting IL-33 in autoimmunity and inflammation. *J. Pharmacol. Exp. Ther.* 354 24–31. 10.1124/jpet.114.222505 25906776

[B142] Theoharides T. C. Stewart J. M. Hatziagelaki E. Kolaitis G. (2015b). Brain “fog,” inflammation and obesity: key aspects of neuropsychiatric disorders improved by luteolin. *Front. Neurosci.* 9:225. 10.3389/fnins.2015.00225 26190965 PMC4490655

[B143] Theoharides T. C. Tsilioni I. Arbetman L. Panagiotidou S. Stewart J. M. Gleason R. M. (2015c). Fibromyalgia, a syndrome in search of pathogenesis and therapy. *J. Pharmacol. Exp. Ther.* 355 255–263. 26306765 10.1124/jpet.115.227298 PMC4613957

[B144] Theoharides T. C. Valent P. Akin C. (2015d). Mast cells, mastocytosis, and related disorders. *N. Engl. J. Med.* 373 163–172. 10.1056/nejmra1409760 26154789

[B145] Theoharides T. C. Stewart J. M. Tsilioni I. (2017). Tolerability and benefit of a tetramethoxyluteolin-containing skin lotion. *Int. J. Immunopathol. Pharmacol.* 30 146–151. 10.1177/0394632017707610 28480804 PMC5806797

[B146] Theoharides T. C. Tsilioni I. (2018). Tetramethoxyluteolin for the treatment of neurodegenerative diseases. *Curr. Top. Med. Chem.* 18 1872–1882. 10.2174/1568026617666181119154247 30451113

[B147] Theoharides T. C. Tsilioni I. Ren H. (2019). Recent advances in our understanding of mast cell activation - or should it be mast cell mediator disorders? *Expert. Rev. Clin. Immunol.* 15 639–656. 10.1080/1744666X.2019.1596800 30884251 PMC7003574

[B148] Thonhoff J. R. Simpson E. P. Appel S. H. (2018). Neuroinflammatory mechanisms in amyotrophic lateral sclerosis pathogenesis. *Curr. Opin. Neurol.* 31 635–639. 10.1097/WCO.0000000000000599 30048339

[B149] Torresani C. Bellafiore S. De Panfilis G. (2009). Chronic urticaria is usually associated with fibromyalgia syndrome. *Acta Derm. Venereol.* 89 389–392. 10.2340/00015555-0653 19688152

[B150] Tsilioni I. Panagiotidou S. Theoharides T. C. (2014). Exosomes in neurologic and psychiatric disorders. *Clin. Ther.* 36 882–888. 10.1016/j.clinthera.2014.05.005 24913029

[B151] Tsilioni I. Russell I. J. Stewart J. M. Gleason R. M. Theoharides T. C. (2016). Neuropeptides CRH, SP, HK-1, and inflammatory cytokines IL-6 and TNF are increased in serum of patients with fibromyalgia syndrome, implicating mast cells. *J. Pharmacol. Exp. Ther.* 356 664–672. 10.1124/jpet.115.230060 26763911 PMC4767394

[B152] Tsilioni I. Theoharides T. C. (2018). Extracellular vesicles are increased in the serum of children with autism spectrum disorder, contain mitochondrial DNA, and stimulate human microglia to secrete IL-1beta. *J. Neuroinflammation* 15:239. 10.1186/s12974-018-1275-5 30149804 PMC6112123

[B153] Uceyler N. Hauser W. Sommer C. (2011). Systematic review with meta-analysis: cytokines in fibromyalgia syndrome. *BMC Musculoskelet Disord.* 12:245. 10.1186/1471-2474-12-245 22034969 PMC3234198

[B154] Vargas D. L. Nascimbene C. Krishnan C. Zimmerman A. W. Pardo C. A. (2005). Neuroglial activation and neuroinflammation in the brain of patients with autism. *Ann. Neurol.* 57 67–81. 10.1002/ana.20315 15546155

[B155] Wallon C. Yang P. Keita A. V. Ericson A. C. McKay D. M. Sherman P. M. (2008). Corticotropin releasing hormone (CRH) regulates macromolecular permeability via mast cells in normal human colonic biopsies in vitro. *Gut* 57 50–58. 10.1136/gut.2006.117549 17525093

[B156] Wang W. Ji P. Riopelle R. J. Dow K. E. (2002). Functional expression of corticotropin-releasing hormone (CRH) receptor 1 in cultured rat microglia. *J. Neurochem.* 80 287–294. 10.1046/j.0022-3042.2001.00687.x 11902119

[B157] Wolfe F. Walitt B. (2013). Culture, science and the changing nature of fibromyalgia. *Nat. Rev. Rheumatol.* 9 751–755. 10.1038/nrrheum.2013.96 23820862

[B158] Woodman I. (2013). Fibromyalgia: fibromyalgia-all in the brain? *Nat. Rev. Rheumatol.* 9:565. 10.1038/nrrheum.2013.137 24045710

[B159] Woolf C. J. (2011). Central sensitization: implications for the diagnosis and treatment of pain. *Pain* 152 S2–S15. 10.1016/j.pain.2010.09.030 20961685 PMC3268359

[B160] Wu Y. Dissing-Olesen L. MacVicar B. A. Stevens B. (2015). Microglia: dynamic mediators of synapse development and plasticity. *Trends Immunol.* 36 605–613. 10.1016/j.it.2015.08.008 26431938 PMC4841266

[B161] Xanthos D. N. Gaderer S. Drdla R. Nuro E. Abramova A. Ellmeier W. (2011). Central nervous system mast cells in peripheral inflammatory nociception. *Mol. Pain* 7:42. 10.1186/1744-8069-7-42 21639869 PMC3123586

[B162] Yunus M. B. (2007). Fibromyalgia and overlapping disorders: the unifying concept of central sensitivity syndromes. *Semin. Arthritis Rheum.* 36 339–356. 10.1016/j.semarthrit.2006.12.009 17350675

[B163] Zhang B. Asadi S. Weng Z. Sismanopoulos N. Theoharides T. C. (2012). Stimulated human mast cells secrete mitochondrial components that have autocrine and paracrine inflammatory actions. *PLoS One* 7:e49767. 10.1371/journal.pone.0049767 23284625 PMC3524249

[B164] Zhang S. Zeng X. Yang H. Hu G. He S. (2012). Mast cell tryptase induces microglia activation via protease-activated receptor 2 signaling. *Cell Physiol. Biochem.* 29 931–940. 10.1159/000171029 22613992

[B165] Zhang Q. Raoof M. Chen Y. Sumi Y. Sursal T. Junger W. (2010). Circulating mitochondrial DAMPs cause inflammatory responses to injury. *Nature* 464 104–107. 10.1038/nature08780 20203610 PMC2843437

[B166] Zhang X. Wang Y. Dong H. Xu Y. Zhang S. (2016). Induction of microglial activation by mediators released from mast cells. *Cell Physiol. Biochem.* 38 1520–1531. 10.1159/000443093 27050634

[B167] Zhang Z. Cherryholmes G. Mao A. Marek C. Longmate J. Kalos M. (2008). High plasma levels of MCP-1 and eotaxin provide evidence for an immunological basis of fibromyalgia. *Exp. Biol. Med.* 233 1171–1180. 10.3181/0712-RM-328 18535166

